# Occupational health patients’ parallel use of primary- and secondary-care services and linkage to work disability: A follow-up study in Finland

**DOI:** 10.1177/14034948221130438

**Published:** 2022-11-02

**Authors:** Tiia Reho, Salla Atkins, Mikko Korhonen, Anna Siukola, Mervi Viljamaa, Markku Sumanen, Jukka Uitti, Riitta Sauni

**Affiliations:** 1 Faculty of Medicine and Health Technology, Tampere University, Finland; 2 Pihlajalinna Työterveys, Finland; 3 Department of Global Public Health, Karolinska Institutet, Sweden; 4 New Social Research and Faculty of Social Sciences, Tampere University, Finland; 5 Clinic of Occupational Medicine, Tampere University Hospital, Finland

**Keywords:** Occupational health services, primary health care, health-care utilisation, working age, work disability, work ability, sick leave, insurance medicine, intersectoral collaboration

## Abstract

**Aims:**

This study aimed to investigate occupational health (OH) primary-care patients’ use of other health-care services and whether parallel use affects their likelihood to have sickness absences (SA) or disability pensions (DP).

**Methods:**

Primary-care services in Finland are provided through three parallel health-care sectors, all available to the working population: public, private and OH sectors. Patients may also be referred to secondary care. This follow-up study combines real-world medical record data containing SA data from a nationwide OH provider with health-care attendance data from public and private primary-care sectors and public secondary care, sociodemographic data and DP decisions. Patients between 18 and 68 years of age who used OH primary care at least once during the study years 2014–2016 were included. The total study population comprised 59,650 patients. Odds ratios were used to analyse association between parallel service use and SA or DP.

**Results:**

Females and patients with a lower educational level were more likely to use services in other health-care sectors in addition to OH than others. Those patients who used any other health-care sector in addition to OH primary care had an increased likelihood of having long SA or receiving DP.

**Conclusions:**

**OH primary-care patients using the services of several health-care sectors in parallel have an increased likelihood of receiving disability benefits – either SA or DP. There is need for care coordination to ensure adequate measures for work-ability support.**

## Background

Disability pensions (DP) that cause both personal loss and social challenges cost €2.4 billion in Finland in 2019 [1]. Joint efforts of different health-care sectors are needed to prevent unnecessary early retirement and lost working days due to sickness absence (SA). Concurrently, health-care costs keep rising, particularly in secondary care, adding pressure to managing health-care costs [2]. It is assumed that coordination could lower health-care costs and prevent work disability more effectively.

Given their close contact with the workplace, occupational health services (OHS) should be best placed to support work ability and remaining in the workforce. Work disability rates may increase when a health condition is not identified early and timely information on treatment, prognosis and work accommodation possibilities is not shared. Other service sectors than OHS are not linked to workplaces’ return-to-work programmes and may not know of rehabilitative possibilities and feel uneasy in determining work ability [3,4]. This may lead to suggesting DP too early and as the only possibility.

Primary health care is provided through three health-care sectors in Finland: public, private and occupational health (OH) [5]. All three sectors are available for 90% of the working population, allowing several options for using health services [6,7]. Using the services of several health-care sectors may lead to scattering of care and either overlapping treatment or lack of information about supportive measures. Patients, on the other hand, value the opportunity to make choices in health care [8]. Among the few studies examining this phenomenon in Finland, survey studies have suggested that if available, OH primary care is often used as the sole primary-care provider [6,7].

Frequent health-service use is associated with long SA and DP [9,10], but less is known of the effect of using several health-care sectors in parallel. Is there a greater risk of work disability when treatment is delivered in several health-care sectors that rarely communicate with each other? When using several health-care sectors, there is a risk of losing vital information on the illness and the care plan because information is not easily transferred across different sectors’ electronic medical record systems [11]. Additionally, information on work and work ability is available infrequently in health records [12]. Thus, transferring information may rely only on the patients, and coordination of care could be beneficial.

OHS is financed for the most part by employers, and employees contribute through an insurance plan collected from both employers and employees. The Finnish OH has two roles: mandatory preventive actions and voluntary primary care, arranged by the same service provider [13,14]. Public primary- and secondary-care services are available to all citizens with a co-payment, while most costs are subsidised by taxes. Private primary care is paid for by the user, and a minor compensation is subsidised through the Social Insurance Institution of Finland (KELA). Patients may choose to use one or all service sectors at primary-care level. Most secondary care, including outpatient and inpatient episodes, is organised publicly and available after a referral.

OHS has a statutory role and offers the best expertise in rehabilitative measures to support work ability and the possibility of discussing work accommodations with employers in Finland [15–17]. Thus, when treating the working population, OHS should be integrated in the care plan as the expert in work ability and rehabilitation. However, patients might choose to use other service sectors. In order to examine patients’ use of health-care sectors and to understand how care coordination could be improved, we first need to understand if patients are using other health-care sectors while they are clients of OHS. We also need to examine whether this is linked to SA or DP.

### Aims

This study aimed to examine how patients in OH primary care use parallel health-care services (primary and secondary) and whether use of several service sectors is associated with the likelihood of having SA and DP.

## Methods

### Study setting and design

This is a follow-up study combining medical record data from Pihlajalinna with comprehensive register data from several data owners. Pihlajalinna data were used to form the study population and also, for SA data, to acquire the short SA not available through other registers. Pihlajalinna is a large OH provider functioning in both urban and rural areas of Finland, and at the time of this study, it included 40 OH units. Visits to both private and public health services were acquired from the Finnish Institute for Health and Welfare (THL) and KELA. The data were completed with sociodemographic data from Statistics Finland and DP decisions from the Finnish Centre for Pensions (FCP).

In Finland, DP may be granted if a patient’s work ability is impaired due to an illness for a period of more than a year. Partial DP may be granted if work ability is reduced by two fifths, and full DP if work ability is reduced is at least three fifths. Both benefits may be granted either permanently or for a fixed term if rehabilitation is expected. DP are funded through mandatory insurance paid by all employees and employers.

### Data collection and study population

Data from Pihlajalinna included visits to different professionals and SA during 2014–2016. These data were sent to Statistics Finland, which pseudonymised the data and added sociodemographic data from their FOLK database [18]. Visits to public primary care and public secondary care and their diagnostic codes received from THL’s registers were combined with the data from Statistics Finland. We included only outpatient visits from public secondary care. Data on visits to private health care were collected from KELA, and data on DP were collected from the FCP, and both were added to the data set. Tampere University processed the pseudonymised data in the information safe online environment provided by Statistics Finland.

Our initial data comprised 78,507 patients. The study material was limited to employees aged 18–68 years who had visited OH primary care at least once during the study years 2014–2016. All patients who had used OH primary care at least once during these years were included. Only illness-related visits conducted face to face were included. Health check-ups that were not initiated by the patient were excluded. After exclusions, our study comprised 59,650 patients. A flow diagram of patient categorisation and exclusions can be found in a previous paper [9].

### Statistical analysis

The descriptive statistics included distribution of different occupational classes, educational level, living alone and employer size in 2015. We examined the association between employees’ sociodemographic factors and employer characteristics with using parallel services through multinomial logistic regression. Multinomial logistic regression was used to analyse whether parallel health-care use was associated with SA (short 1–3 days, intermediate 4–14 days or long ⩾15 days) in 2014–2016 and DP (partial fixed-term DP, partial DP, fixed-term DP and permanent DP) granted between 2017 and 2018 when compared to those patients who did not use health-care sectors other than OHS. In the adjusted models, we adjusted for sex, age, occupational class, educational level, living alone and employer’s size and industry and unemployment. The data were analysed using R (The R Foundation for Statistical Computing, Vienna, Austria), and *p*-values of <0.05 were considered statistically significant.

## Results

The study population comprised 59,650 patients who had used OH primary-care services during 2014–2016. There were more males than females in the group that had not visited health-care sectors other than OH primary care (Table I).

**Table I.** Descriptive data of occupational health primary-care patients having visited other health-care sectors (2014–2016), *N*=59,650.

	Having visited no other health-care sectors, *N*=8869		Having visited private primary care, *N*=30,328		Having visited public primary care, *N*=33,580		Having visited public secondary care, *N*=30,584		Having visited all three other health-care sectors, *N*=11,504	
	*n*	%	*n*	%	*n*	%	*n*	%	*n*	%
Sex										
Male	7080	80	13713	45	17070	51	15841	52	4534	39
Female	1789	20	16615	55	16510	49	14743	48	6970	61
Age (years)										
18–34	2948	33	8273	27	12133	36	9895	32	3605	31
35–44	2364	27	6858	23	7283	22	6620	22	2296	20
45–54	2279	26	8047	27	7522	22	7341	24	2680	23
55–68	1278	14	7150	24	6642	20	6728	22	2923	25
Educational level										
High	3589	41	14249	47	11425	34	11532	38	4562	40
Intermediate	4195	48	13371	44	18118	54	15530	51	5699	50
Low	896	10	2617	9	3919	12	3396	11	1219	11
Occupational class										
Upper non-manual	2067	24	6470	21	4618	14	5023	16	1775	15
Lower non-manual	2270	26	10330	34	10514	31	9775	32	3923	34
Manual	3138	36	7589	25	10803	32	9443	31	3069	27
Entrepreneurs	331	4	1550	5	1169	3	1190	4	497	4
Others	874	10	4298	14	6358	19	5027	17	2216	19
Living alone										
Alone	1714	20	5552	19	6353	19	5861	19	2135	19
Not alone	6883	80	24414	81	26732	81	24272	81	9219	81
Employer size										
1–10	1218	15	4463	15	4914	15	4341	15	1711	16
11–50	2490	30	7751	27	9357	29	8165	28	3003	27
51–250	2235	27	7288	25	8235	26	7373	26	2697	25
>250	2294	28	9392	33	9483	30	8902	31	3584	33

Public secondary-care services were used by 10% of OH primary-care patients during all of the three study years (Table II). The proportions having used public primary care and private primary care in all three study years were 11% and 13%.

**Table II.** Proportion of OH primary-care patients having visited the different health-care sectors in 2014–2016, *N*=59,650.

OH primary-care patients 2014–2016	*n* (%)	*n* (%)	*n* (%)	*n* (%)
Private primary care	No private primary-care visits	Private primary-care visits in one year	Private primary-care visits in two to three years	Private primary-care visits in all three years
	29,322 (49%)	14,914 (25%)	9101 (15%)	6313 (11%)
Public primary care	No public primary-care visits	Public primary-care visits in one year	Public primary-care visits in two to three years	Public primary-care visits in all three years
	26,070 (44%)	15,586 (26%)	10,502 (18%)	7492 (13%)
Public secondary care	No public secondary-care visits	Public secondary-care visits in one year	Public secondary-care visits in two to three years	Public secondary-care visits in all three years
	29,066 (49%)	15,495 (26%)	8910 (15%)	6179 (10%)

OH: occupational health.

Female sex was associated with visits to all health-care sectors other than OHS (Table III). Intermediate or low educational level was associated with visits to public primary and secondary care. Entrepreneurs were likely to use both private and public primary care.

**Table III.** Predictive value of OH primary-care patients’ sociodemographic characteristics for parallel service use in 2014–2016, *N*=59,650.

	Having visited private primary care, *N*=30,328		Having visited public primary care, *N*=33,580		Having visited public secondary care, *N*=30,584		Having visited all three other health-care sectors, *N*=11,504	
	OR	95% CI	OR	95% CI	OR	95% CI	OR	95% CI
Sex								
Male	Ref.		Ref.		Ref.		Ref.	
Female	2.95 (2.d33.07)	2.84–3.07	1.71	1.64–1.78	1.48	1.43–1.54	2.37	2.25–2.48
Age (years)								
18–34	Ref.		Ref.		Ref.		Ref.	
35–44	1.18	1.13–1.24	0.79	0.75–0.83	0.92	0.88–0.96	0.92	0.87–0.98
45–54	1.46	1.39–1.53	0.67	0.64–0.7	0.96	0.92–1.01	0.97	0.92–1.03
55–68	1.96	1.86–2.07	0.78	0.74–0.82	1.25	1.19–1.31	1.38	1.3–1.46
Educational level								
High	Ref.		Ref.		Ref.		Ref.	
Intermediate	0.74	0.71–0.77	1.49	1.42–1.55	1.18	1.13–1.23	1.07	1.01–1.13
Low	0.62	0.58–0.66	1.60	1.49–1.71	1.27	1.19–1.35	1.06	0.98–1.15
Occupational class								
Upper non-manual	Ref.		Ref.		Ref.		Ref.	
Lower non-manual	0.84	0.79-0.88	1.27	1.2–1.34	1.11	1.05–1.17	1.06	0.99–1.14
Manual	0.74	0.7–0.79	1.55	1.45–1.65	1.15	1.08–1.22	1.13	1.04–1.22
Entrepreneurs	1.56	1.41–1.74	1.14	1.03–1.26	1.08	0.98–1.19	1.34	1.18–1.52
Others	0.94	0.87–1.01	2.24	2.08–2.41	1.4	1.3–1.49	1.60	1.47–1.74
Living alone								
Alone	1	0.96–1.05	0.89	0.86–0.94	1.02	0.97–1.06	0.95	0.9–1.01
Not alone	Ref.		Ref.		Ref.		Ref.	
Employer size								
1–10	0.87	0.82-0.93	1.18	1.1–1.25	1.09	1.02–1.16	1.06	0.98–1.14
11–50	0.8	0.76-0.84	1.07	1.02–1.13	1.03	0.98–1.08	0.95	0.89–1.01
51–250	0.83	0.78-0.87	1.05	0.996–1.1	1.02	0.97–1.07	0.93	0.87–0.98
>250	Ref.		Ref.		Ref.		Ref.	

Adjusted for other health-care sectors (primary care, public primary care, secondary care) and for other sociodemographic factors (sex, age, educational level, occupational class, employer size and industry, unemployment and living alone).

OR: odds ratio; CI: confidence interval; Ref.: reference group=1.0.

Patients using public or private primary care were more likely to have a SA episode of >15 days compared to patients who used no health-care sector other than OHS (Table IV). The association of long SA was strongest when having visited public secondary care in addition to OHS.

**Table IV.** Predictive value of parallel service use by OH primary care users during 2014–2016 for sickness absence episode of different length in 2014–2016.

	Public primary care	Public secondary care	Private primary care
Short SA (1–3 days)			
Unadjusted	1.28 (1.24–1.32)	1.17 (1.13–1.21)	0.86 (0.83–0.89)
Adjusted, model 1	1.24 (1.2–1.28)	1.12 (1.09–1.16)	0.85 (0.82–0.87)
Adjusted, model 2	1.08 (1.04–1.12)	1.14 (1.1–1.18)	0.93 (0.9–0.97)
Intermediate SA (4–14 days)			
Unadjusted	1.45 (1.4–1.51)	1.65 (1.59–1.71)	1.04 (1.05–1.08)
Adjusted, model 1	1.30 (1.26–1.35)	1.55 (1.49–1.6)	0.99 (0.96–1.03)
Adjusted, model 2	1.19 (1.14–1.24)	1.52 (1.46–1.58)	1.09 (1.05–1.14)
Long SA (⩾15 days)			
Unadjusted	2.09 (1.96–2.22)	4.37 (4.07–4.68)	1.50 (1.42–1.59)
Adjusted, model 1	1.55 (1.45–1.65)	3.83 (3.57–4.12)	1.35 (1.27–1.43)
Adjusted, model 2	1.55 (1.44–1.66)	3.74 (3.47–4.04)	1.31 (1.23–1.4)

Data shown as OR (95% CI). 1.0 (reference group): having used no other health-care sector other than occupational health. Model 1: Adjusted for other health-care sectors (primary care, public primary care, public secondary care). Model 2: Adjusted for other health-care sectors (primary care, public primary care, public secondary care) and for sociodemographic factors (sex, age, educational level, occupational class, employer size and industry and living alone in 2015).

SA: sickness absence.

Using a health-care sector other than OHS increased the likelihood of receiving a DP decision by more than threefold. The perceived association was strongest with public secondary care and weakest with private primary care (Table V). There were 574 patients who received a DP in the study years, and from those, only 21 (4%) had not used any health-care sector besides OH.

**Table V.** Predictive value of parallel service use by OH primary-care users during 2014–2016 for any disability pension decision in 2017–2018.

	DP decision	*n* (% of the study population)	Unadjusted	Adjusted, model 1	Adjusted, model 2
Public primary care	No	142 (0.5)	2.38 (1.97–2.88)	1.70 (1.4–2.07)	1.72 (1.39–2.13)
	Yes	432 (1.3)			
Public secondary care	No	86 (0.3)	5.46 (4.34–6.88)	4.64 (3.67–5.87)	3.85 (3.03–4.89)
	Yes	488 (1.6)			
Private primary care	No	213 (0.7)	1.65 (1.39–1.95)	1.46 (1.23–1.73)	1.30 (1.08–1.56)
	Yes	361 (1.2)			
All other health-care sectors	No	323 (0.7)	3.28 (2.78–3.87)		2.77 (2.32–3.31)
	Yes	251 (2.2)			
Any other health-care sector	No	21 (0.2)	4.55 (2.94–7.04)		3.49 (2.25–5.42)
	Yes	553 (1.1)			

Data shown as OR (95% CI). 1.0 (reference group): having used no other health-care sector. Model 1: Adjusted for other health-care sectors (primary care, public primary care, public secondary care). Model 2: Adjusted for other health-care sectors (primary care, public primary care, public secondary care) and for sociodemographic factors (sex, age, educational level, occupational class, employer size and industry, unemployment and living alone in 2015).

DP: disability pension.

## Discussion

OH primary-care users were likely to use other health-care sectors too. Use of any other health-care sector was associated with the likelihood of having long SA and receiving DP. This association remained when adjusting for sociodemographic factors. Use of public secondary care appeared to be the dominant factor in the association with both SA and DP.

Women are known to become frequent users of health care and use health-care services more often than men [19–21], but to our knowledge, there are no previous data on employed women using multiple health-care sectors in parallel – an association shown in this study. These are probably manifestations of the same phenomenon. A previous study in OH primary care indicated that age was significantly associated with frequent attendance [21], but in this study, older age was associated with the likelihood of using private primary care and, for the oldest age group, also with using secondary care. Use of private primary care might be associated with older employees being in a better economic position than younger employees, while use of public secondary care may be associated with more serious illnesses needing hospital consultations.

The finding that one in four OH primary-care patients use public or private primary care in one year suggests that despite access to OH primary care, there is a need for additional or complementary services. There may be several reasons for this. OH primary care may concentrate on illnesses affecting work ability, and follow-up care of chronic illnesses (e.g. diabetes) might be steered to public primary care. Additionally, despite access to OH primary care, the employer’s contract might limit the availability of more expensive examinations, creating a need for complementary services.

There might also be other reasons for choosing private or public primary care, such as distance, aiming to maintain a long patient–physician relationship, satisfaction with visits and other factors that cannot be accounted for in register studies [22]. Whether patients choose to treat some illnesses with OH and some with other sectors or whether the scattering of care is created by the system is not known. An acknowledged challenge is that other health-care sectors do not have access to OH agreements, and they might not know if OH primary care is available to the patient.

Use of several service sectors might also lead to overlapping examinations and treatments. In return, this may lead to unnecessary costs in health care that could be avoided through careful planning. However, use of several service sectors might also be planned and appropriate if patients have chronic illnesses that need specialist care. Whenever the possibility of using parallel services exists and if the patient suffers from chronic illnesses, there should perhaps be a coordinating sector – for working patients in Finland, preferably the OHS. A plan to indicate where and by whom different aspects of care are covered should make sure that possible work-ability issues are steered to OHS. A coordinated view on the past and planned care should be available to all health-care sectors. At present, electronic health records do not support this aim [23]. Information is often transferred solely through the patient, and health-care professionals might assume that issues are handled elsewhere, leading to inadequate treatment.

Previous studies have shown that frequent use of services is associated with SA of all lengths and in particular long SA [10,24]. On the other hand, SA are associated with unfavourable economic conditions, unemployment and further disability [25,26]. Our study adds to this information by showing the association of the use of several health-care sectors and SA – in particular long SA – even when adjusting for income, occupational status and other sociodemographic factors. There might be several possible explanations for this. Patients who need public secondary-care services are likely to suffer from more severe illnesses, which may involve long SA. Moreover, patients who have challenges concerning work ability possibly seek help from multiple sources. However, there could also be reasons such as physicians in other health-care sectors not being aware of the possibilities of modifying work and rehabilitative possibilities and feelings of inadequacy in determining work ability [3,4]. Whatever the underlying reasons are, this emphasises the need for evaluation of work ability and necessary support measures when a patient is treated by multiple health-care sectors.

To our knowledge, this study is the first to examine the association between multiple health-care sector use and DP through register data. Work disability is an recognised problem in industrialised countries. Governments underline the importance of ensuring that workers do not leave the labour market prematurely for health reasons [27,28], as illness-based retirement is not only a loss to the individual but also an economic and social challenge. According to our study, OH primary-care patients using several health-care sectors are at increased risk of receiving DP. A possible explanation could be that the work disability risks of patients who use several health-care sectors may not be identified in a timely manner, and they might not receive the supportive interventions they need. Without coordination of care and services, and a perspective on treatment delivered in other health-care sectors, OHS cannot support the work ability of the employees. Notably, the use of public secondary care appears to have the greatest effect on DP risk, which was expected, as patients suffering from severe illnesses are referred to secondary care.

In systems with parallel health-care sectors, referral systems should be available across all service sectors. Additionally, work-related illnesses and work disability risks should be identified in all health-care sectors and steered to OH. There is a need for education and mechanisms that support steering patients to OH in these cases. Currently, there are no widely used mechanisms to guide patients in need of OH care to their service provider. A case manager with a view of all health-care records could be a solution when care is scattered across multiple sectors. Communication between service sectors is needed to make sure work-ability issues are not being overlooked or missed.

OHS has a statutory role in the coordination of return-to-work programmes, work-ability support and rehabilitation [16,29]. OHS works in close contact with the workplace and probably has the best knowledge on the possibilities of the social security system to support return to work. Vocational rehabilitation when people are unable to continue in their previous job may be an alternative to DP. OH has several ways to support work ability and return to work, such as job accommodation [30] that might allow patients suffering from musculoskeletal disorders and mental-health disorders [31] to continue working. A case manager in the OHS could also add a rehabilitation perspective to the individual care plan and make sure no overlapping services are being offered.

A strength of the study is the combination of OH real-world data with multiple good-quality registers. Given the comprehensive sociodemographic data available, adjusting for confounding factors was possible. Using the data of a nationwide OH provider is likely to reduce the effect of external factors such as differences in practices and geographical distances. Use of record data and the large study sample dilute human error and recall bias. The findings of the study can be generalised to the working population in Finland to some extent. Our findings could be indicative of similar risks in other populations where parallel services are used. It should be noted that in health-care systems that are organised very differently, there might be different kinds of needs and the health-care sector that should coordinate care could be one other than the OHS. However, these findings emphasise the need for communication and information sharing between all sectors taking part in the treatment.

However, the study has limitations. We have used solely outpatient visits to public secondary care and excluded inpatient episodes. We could not control for changes in occupational status during the study years. Thus, patients might use other service sectors more because they do not have access to OH care. Another limitation is the lack of data on the comprehensiveness of OH contracts which might affect the need to use other service sectors. However, 90% of the working population has access to OH primary care [32]. OH allows visits to physicians and nurses but might limit use of more refined laboratory tests or imaging. This register study could not account for patients’ personal reasons to choose a certain service sector such as distance to the service provider or their diagnoses and their severity which might affect the use of health-care services. Despite the limitations, given that this is the first study to examine the association of multisectoral health-care use and work disability, this study sheds light on a very practical and everyday challenge of a clinician’s work, and we think it has value to the public health audience.

## Conclusions

Patients of OH primary care who use several health-care sectors have an increased likelihood of having SA and receiving DP. The association was highlighted with visits in public secondary care. Care coordination is vital to patients using parallel health-care sectors to manage and prevent work disability. The coordination of care should be strengthened, and medical record systems should support information transfer across health-care sectors. In systems with parallel health-care sectors, referral systems should be available from all service sectors to another. Additionally, work-related illnesses and work disability risks should be identified in all health-care sectors and steered to OHS.

Further research is needed on whether OHS’s interventions and care coordination initiatives could reduce work disability with patients using parallel services. It is also necessary to examine patients’ perspectives on why they choose a certain service provider and which illnesses are treated in multiple sectors.

## References

[1] Pension recipients in Finland 2019. Finnish Centre for Pensions. Helsinki: Official Statistics of Finland. https://www.julkari.fi/bitstream/handle/10024/140540/statistical-yearbook-of-pensioners-in-finland-2019.pdf?sequence=1&isAllowed=y (2020).

[2] Terveydenhuollon menot ja rahoitus 2018 [Health Expenditure and Financing 2018] (In Finnish). Statistical Report 23/2020. Helsinki: Official Statistics of Finland, 2020.

[3] Hinkka Hinkka K Niemelä M Autti-Rämö I . Sairauspoissaolotarpeen määrittäminen. Kyselytutkimus lääkäreille [Determining sickness absences. A survey study]. Kela. Työpapereita 96/2016. http://hdl.handle.net/10138/163080

[4] Ljungquist T Hinas E Nilsson GH , et al. Problems with sickness certification tasks: experiences from physicians in different clinical settings. A cross-sectional nationwide study in Sweden. BMC Health Serv Res 2015;15:1–10. 26264627 10.1186/s12913-015-0937-6 PMC4533961

[5] Ministry of Social Affairs and Health. Health care in Finland. Brochures of the Ministry of Social Affairs and Health 2eng (2013). Helsinki. http://www.urn.fi/URN:ISBN:978-952-00-3395-8 (2013).

[6] Virtanen P Mattila K . Työterveyslääkärin potilas käy myös terveyskeskuksessa, tosin harvoin [Patients of occupational health physicians also visit health centre GPs, albeit seldom]. Suom Lääkäril 2011;47:3583–6.

[7] Ikonen A Räsänen K Manninen P , et al. Use of health services by Finnish employees in regard to health-related factors: the population-based Health 2000 study. Int Arch Occup Environ Health 2013;86:451–62. 10.1007/s00420-012-0778-0 22562521

[8] Aalto AM Elovainio M Tynkkynen LK , et al. What patients think about choice in healthcare? A study on primary care services in Finland. Scand J Public Health 2018;46:463–70. 10.1177/1403494817731488 28925813

[9] Reho TTM Atkins SA Talola N , et al. Frequent attenders at risk of disability pension: a longitudinal study combining routine and register data. Scand J Public Health 2020;48:181–9. 10.1177/1403494819838663 PMC7042497 30973068

[10] Perhoniemi R Blomgren J . Frequent attenders of three outpatient health care schemes in Finland: characteristics and association with long-term sickness absences, 2016–2018. BMC Public Health 2021;21:1–14. 33957897 10.1186/s12889-021-10866-x PMC8101095

[11] Saastamoinen P Hyppönen H Kaipio J , et al. Lääkärien arviot potilastietojärjestelmistä ovat parantuneet hieman [Slight positive changes in physicians’ assessments of electronic health record systems]. Suom Lääkäril 2018;34:1814–9.

[12] Nissinen S Oksanen T Kinnunen U-M , et al. Työkykyä koskeva tieto työterveyshuollon tietojärjestelmissä [Work ability data in electronic health records of occupational health services]. Suom Lääkäril 2017;72:2013–7b.

[13] Vuorenkoski L Mladovsky P Mossialos E . Finland: Health system review. Geneva: World Health Organization, Regional Office for Europe, European Observatory on Health Systems and Policies, 2008.

[14] Asetus hyvästä työterveyshuoltokäytännöstä. 708/2013. Government decree on the principles of good occupational health practice, the content of occupational health care and the educational qualifications required of professionals and experts. (In Finnish) https://www.finlex.fi/fi/laki/alkup/2013/20130708.

[15] Lappalainen L Liira J Lamminpää A , et al. Work disability negotiations: supervisors’ view of work disability and collaboration with occupational health services. Disabil Rehabil 2019;41:2015–25. 10.1080/09638288.2018.1455112 29587552

[16] Occupational Health Care Act. Työterveyshuoltolaki. 1383/2001.

[17] Työterveys 2025 – Yhteistyöllä työkykyä ja terveyttä [Occupational Health 2025 – Work ability and Health through collaboration] (In Finnish). Valtioneuvoston periaatepäätös. http://urn.fi/URN:ISBN:978-952-00-3799-4 (2017).

[18] Statistics Finland. FOLK database, https://taika.stat.fi/en/aineistokuvaus.html#!?dataid=FOLK_19872019_jua_perus20_002.xml (accessed 30 March 2021).

[19] Neal RD Heywood PL Morley S , et al. Frequency of patients’ consulting in general practice and workload generated by frequent attenders: comparisons between practices. Br J Gen Pract 1998;48:895–8. PMC1409909 9604412

[20] Gill D Sharpe M . Frequent consulters in general practice: a systematic review of studies of prevalence, associations and outcome. J Psychosom Res 1999;47:115–30. 10.1016/s0022-3999(98)00118-4 10579496

[21] Reho T Atkins S Talola N , et al. Frequent attenders in occupational health primary care – a cross-sectional study. Scand J Public Health 2019;47:28–36. 29806549 10.1177/1403494818777436

[22] Oksanen K Sauni R Koskinen A , et al. Työikäisten arviot avohoidon lääkärissäkäynneistä [Satisfaction with and perceived benefit of the latest physician visit among working-aged Finns]. Suom Lääkäril 2015;42:2777–2784.

[23] Kaipio J Lääveri T Hyppönen H , et al. Usability problems do not heal by themselves: national survey on physicians’ experiences with EHRs in Finland. Int J Med Inform 2017;97:266–81. 10.1016/j.ijmedinf.2016.10.010 27919385

[24] Reho T Atkins S Talola N , et al. Occasional and persistent frequent attenders and sickness absences in occupational health primary care: a longitudinal study in Finland. BMJ Open 2019;9:e024980. 10.1136/bmjopen-2018-024980 PMC6411255 30782922

[25] Bryngelson A . Long-term sickness absence and social exclusion. Scand J Public Health 2009;37:839–45. 10.1177/1403494809346871 19726527

[26] Virtanen M Kivimäki M Vahtera J , et al. Sickness absence as a risk factor for job termination, unemployment, and disability pension among temporary and permanent employees. Occup Env Med 2006;63:212–7. 10.1136/oem.2005.020297 PMC2078149 16497865

[27] Waddell G Burton KA . Is work good for your health and well-being? London: The Stationery Office, 2006.

[28] Työelämäryhmän loppuraportti. Ehdotuksia työurien pidentämiseksi. [Suggestions to prolong working careers] (In Finnish). https://www.etk.fi/wp-content/uploads/2020/05/tyoelamaryhman-loppuraportti.pdf (2010).

[29] Uitti J , ed. Hyvä työterveyshuoltokäytäntö [Good occupational health practice]. 3rd–4th ed. Helsinki: Työterveyslaitos, 2014. pp.217–8.

[30] Van Oostrom S Driessen M de Vet H , et al. Workplace interventions for preventing work disability. Cochrane Database Syst Rev 2009;1–67. 10.1002/14651858.CD006955.pub2 19370664

[31] Mikkelsen MB Rosholm M . Systematic review and meta-analysis of interventions aimed at enhancing return to work for sick-listed workers with common mental disorders, stress-related disorders, somatoform disorders and personality disorders. Occup Environ Med 2018;75:675–86. 10.1136/oemed-2018-105073 29954920

[32] Lappalainen K Aminoff M Hakulinen H , et al. Työterveyshuolto Suomessa vuonna 2015 [Occupational health care in Finland 2015 report] (In Finnish). Työterveyslaitos. https://urn.fi/URN:ISBN:978-952-261-673-9 (2016)

